# Mycobacterium tuberculosis Transcriptome Profiling in Mice with Genetically Different Susceptibility to Tuberculosis

**Published:** 2013

**Authors:** T.A. Skvortsov, D.V. Ignatov, K.B. Majorov, A.S. Apt, T.L. Azhikina

**Affiliations:** Shemyakin and Ovchinnikov Institute of Bioorganic Chemistry, Russian Academy of Sciences, Miklukho-Maklaya Str., 16/10, Moscow, Russia, 117997; 2Central Institute for Tuberculosis, Yauza Alley, 2, Moscow, Russia, 107564

**Keywords:** Mycobacterium tuberculosis, transcriptome in vivo, next generation sequencing, RNA-seq, tuberculosis

## Abstract

Whole transcriptome profiling is now almost routinely used in various fields of biology, including microbiology. *In vivo *transcriptome studies usually provide relevant information about the biological processes in the organism and thus are indispensable for the formulation of hypotheses, testing, and correcting. In this study, we describe the results of genome-wide transcriptional profiling of the major human bacterial pathogen* M. tuberculosis *during its persistence in lungs*. *Two mouse strains differing in their susceptibility to tuberculosis were used for experimental infection with *M. tuberculosis*. Mycobacterial transcriptomes obtained from the infected tissues of the mice at two different time points were analyzed by deep sequencing and compared. It was hypothesized that the changes in the *M. tuberculosis *transcriptome may attest to the activation of the metabolism of lipids and amino acids, transition to anaerobic respiration, and increased expression of the factors modulating the immune response. A total of 209 genes were determined whose expression increased with disease progression in both host strains (commonly upregulated genes, CUG). Among them, the genes related to the functional categories of lipid metabolism, cell wall, and cell processes are of great interest. It was assumed that the products of these genes are involved in *M. tuberculosis *adaptation to the host immune system defense, thus being potential targets for drug development.

## INTRODUCTION

Genome-wide expression studies generate enormous amounts of data and have a virtually boundless potential for application. For instance, the data obtained in such experiments can be used to assess the effectiveness of antibiotics or to study changes in the bacterial metabolism during the course of an infection. Among the infections caused by pathogenic bacteria, tuberculosis ranks first in terms of mortality rate and is responsible for 1.5 million deaths annually. It is not surprising that the first study of the transcriptome of its causative agent, *Mycobacterium tuberculosis*, was conducted within a year after the publication of the whole-genome sequencing data for this bacterium [1, 2]. Five years later, a large number of studies describing the results of using microarrays for the transcriptome analysis of mycobacteria under various conditions were published [3, 4]. Nevertheless, although microarrays and massively parallel sequencing have been widely used for microbiological studies and adapted to the study of whole-genome expression in mycobacteria, the majority of such studies have been carried out *in vitro*. Meanwhile, it is rather difficult to carry out an analysis of gene expression in mycobacteria during the progression of tuberculosis *in vivo*, which is of the greatest research interest [5, 6]. The results of a study of gene expression in *M. tuberculosis* during experimental infections in two strains of mice differing in susceptibility to tuberculosis are presented in this work. Since the genotypic differences of animals directly affect gene expression of the pathogen, an increase in the expression of a number of bacterial genes under more unfavorable conditions (in the organism of a host resistant to the infection) attests to the fact that these genes significantly contribute to the adaptation of* M. tuberculosis *to the host’s defense mechanisms.

## EXPERIMENTAL PROCEDURES

**Experimental infection and RNA extraction**

I/StSnEgYCit (I/St) and C57BL/6YCit (B6) mice were kept under standard conditions in the vivarium of the Central Research Institute of Tuberculosis, Russian Academy of Medical Science, in accordance with Order of the USSR Ministry of Health № 755 dated August 12, 1977, and the NIH Office of Laboratory Animal Welfare Assurance № A5502-11. The mice had *ad libitum *access to food and water. All the experimental procedures were approved by the Bioethics Committee of the Central Research Institute of Tuberculosis.

Female mice of both strains aged 2.5–3.0 months were infected with the virulent *M. tuberculosis *strain H37Rv using an inhalation exposure system (Glas-Col, Terre Haute, USA) at a dose of 100–200 CFU/mouse. The infected animals were euthanized 4 and 6 weeks after their infection. Their lung tissues were immediately used to extract RN A. Total RN A was extracted using the SV Total RN A Isolation System (Promega, USA). The RN A samples were then treated with DNAse I (MBI Fermentas, Lithuania) to remove trace amounts of DNA.

**Synthesis of cDNA**

cDNA was synthesized using the template-switching effect (Clontech, USA) according to the procedure described in [7]. cDNA was synthesized using PowerScript II reverse transcriptase (Clontech, USA) following the manufacturer’s protocol. The oligonucleotide primers BR (5’AAGCAGTGGTATC AACGCAGAGTAC(N)9) and SMART (5’AAGCAGTGGTATC AACGCAGAGTACGCrGrGrG) were added into 2 μg of total RN A from each sample in 11 μl of a buffer solution. The resulting mixture was incubated for 2 min at 70°С and placed on ice for 10 min. The mixture kept in the ice bath was supplemented with 11 μl of a solution containing 4 μl of a 5× reverse transcriptase buffer, 2 μl of a solution of 100 mM DDT, 2 μl of a solution of 10 mM of each dNT P, and 1 μl (200 AU) of PowerScript reverse transcriptase (Clontech, USA). The control reaction (RT –) with no reverse transcriptase added was conducted simultaneously with the reverse transcriptase reaction (RT +). The reaction mixtures RT + and RT – were incubated at 37°С for 10 min and for an additional 40 min at 42°С. 30 cycles of PCR (95°C 20 s; 64°C, 20 s; 72°C, 2 min) with the 5S primer (5’GTGGTATC AACGCAGAGT) were used to amplify the synthesized cDNA. The amplified cDNA was purified using the QIAquick PCR Purification kit (Qiagen, USA).

**Coincidence cloning**

Coincidence cloning was performed according to [8]. The genomic DNA of *M. tuberculosis *H37Rv and the total cDNA samples (i.e., cDNA synthesized using total RN A as a template) were digested with the restriction endonucleases RsaI and AluI (MBI Fermentas, Lithuania). The suppression adapters were ligated to the resulting fragments of the genomic DNA and cDNA (adapter structures were given in [7]). The mixture containing 100 ng of the sample of genomic DNA with adapters and 100 ng of the corresponding sample of cDNA fragments with adapters in 2 μl of the hybridization buffer HB (50 mM HEPES, pH 8.3; 0.5 M NaCl; 0.02 mM EDTA, pH 8.0) was incubated at 99°C for 5 min (denaturation) and subsequently at 68°C for 18 h (re-naturation). Next, the reaction mixture was supplemented with 100 μl of the hybridization buffer HB preliminarily heated to 68°C. 1 μl of the resulting mixture was used as a template in a two-step PCR . The first PCR step was performed in a reaction volume of 25 μl containing 10 pmol of the outer primer T7. After the mixture was preincubated at 95°C for 5 min, 20 amplification cycles were performed (94°C, 30 s; 66°C, 30 s; 72°C, 90 s). The second amplification step was carried out using inner primers (94°C, 30 s; 68°C, 30 s; 72°C, 90 s; 25 cycles) [7]. The tenfold diluted amplicon obtained in step 1 was used as a template for the second amplification step. The amplicon obtained in step 2 was purified using the QIAquick PCR Purification kit (Qiagen, USA) and used for massively parallel sequencing.

**Massively parallel sequencing**

Prior to sequencing, equal amounts of each of the coincidence cloning products (500 ng of each amplicon) were combined to obtain a single sample. Massively parallel pyrosequencing of the sample was performed on the GS FLX genetic instrument (454 Roche, Germany). The resulting sequences (reads) were tested for the presence of adapter sequences. The reads with truncated, incorrect, or missing adapter sequences were eliminated from further analysis. The remaining sequences (190, 031 reads) were divided into three groups (libraries) depending on the nucleotide sequence of their adapters; СС6(SUS) represented transcriptomes of *M. tuberculosis *from the lung tissues of I/St mice on week 6 post-infection; (СС4(RE S) and СС6(RE S) represented transcriptomes of *M. tuberculosis *from lung tissues of B6 mice on weeks 4 and 6 post-infection, respectively. A file in FASTA format, which contained nucleotide sequences from all three groups, was used for further analysis.

The authors can provide the FASTA files upon request.

**Sequence mapping and statistical analysis**

The resulting sequences were mapped onto the genomic sequence of *M. tuberculosis *H37Rv (assembly GenBank AL123456.2) using the blastn tool from the NC BI BLAST+ software package with the parameters set as follows: -perc identity 95 and -evalue 0.01. The sequences with at least 95% identity to the genome sequence of *M. tuberculosis *H37Rv for the entire length were eventually selected. All the sequences shorter than 40 nucleotides were eliminated from processing. The sequences mapped onto the *M. tuberculosis* genome in a non-unique manner (at two sites and more) were also eliminated from further analysis. The reads mapped onto the intergenic sequences were not eliminated from further analysis, since it would have changed the library size and caused errors during the subsequent statistical analysis. Next, the number of reads in each library was determined for each gene and intergenic region, and the comparative analysis of the abundance of cDNA fragments corresponding to the bacterial genes and intergenic regions was conducted using the Audic-Claverie algorithm [9]. The differences in the expression of the genes (intergenic regions) were considered to be significant if the number of reads mapped onto the gene (intergenic region) in at least one of the two libraries being compared was no less than 20 and *p* < 0.05.

## RESULTS AND DISCUSSION

In order to reveal the features of the expression profile of *M. tuberculosis *that correlate with infection progression, comparative quantitative and qualitative analyses of the sequences transcribed in infected mice, genetically susceptible (inefficient immune response), and resistant (efficient response) to these bacteria were conducted at different stages of the infectious process.

We have compared the transcriptomes of *M. tuberculosis* H37Rv in infected mice of two strains, I/StSn- EgYCit (I/St) and C57BL/6YCit (B6). These mouse strains had been thoroughly described earlier [10]. In B6 mice, resistance to *M. tuberculosis *is higher as compared to I/St mice, which manifests itself in the less aggressive course of the infectious process in B6 mice and the longer survival of the infected animals.

Female mice of both strains were euthanized 4 and 6 weeks after they had been aerogenically infected with* M. tuberculosis *bacteria to isolate the total RN A from their lungs. The total RN A samples from the lung tissues in I/St and B6 mice were used to synthesize cDNA subsequently enriched in bacterial cDNA fragments via coincidence cloning [8]. A total of three sequence libraries representing *M. tuberculosis *transcriptomes were obtained from the tissues of I/St mice on week 6 after the infection (СС6(SUS)) and the tissues of B6 mice on weeks 4 and 6 after they had been infected (СС4(RE S) and СС6(RE S), respectively).

The nucleotide sequences of the cDNA fragments of these libraries were determined using 454 massively parallel pyrosequencing. The general scheme of the experiment is shown in *Figure*; the general characteristics of the analyzed libraries are listed in *Table 1*. The nucleotide sequences of 190, 031 cDNA fragments were identified. Among them, 73, 410 sequences were from the CC 4(RE S) library; 75, 655 were from the CC 6(SUS) library; and 40, 966, from CC 6(RE S). The resulting sequences were mapped onto the genomic sequence of *M. tuberculosis *H37Rv (Assembly GenBank AL123456.2) using the blastn command line tool from the NC BI BLAST+ software package.

**Table 1:** Results of sequencing and library mapping for CC4(RES), CC6(RES), and CC6(SUS)

Library	CC4 (RES)	CC6 (SUS)	CC6 (RES)
Total reads	73410	75655	40966
Mtb-specific reads (unique)	14990	43618	34234
Mtb-specific reads (unique), % of the total number	20.4	57.7	83.6
Genes expressed (number of reads > 0)	1012	1353	1940
Genes expressed, % of the total number of genes	25.2	33.7	48.3
IGR expressed (number of reads > 0)	164	221	376
IGR expressed, % of the total number of genes	5.3	7.2	12.3

The mapping revealed that the CC 4(RE S), CC 6(SUS) and CC 6(RE S) libraries contained 14, 990 (20.42%), 43, 618 (57.65%) and 34, 234 (83.57%) *M. tuberculosis* sequences, respectively. These data attest to the fact that a significant enrichment of cDNA in the bacterial sequences was attained.

Among 4,012 genes and seven pseudogenes of *M. tuberculosis*, 1,012 (25.2% of the total number of genes), 1,353 (33.7%), and 1,940 genes (48.3%) were expressed in the CC 4(RE S), CC 6(SUS), and CC 6(RE S), respectively. 1,428 (35.5%) genes were expressed in none of the samples, while 469 (11.7%) genes were expressed in each sample.

**Fig. 1:**
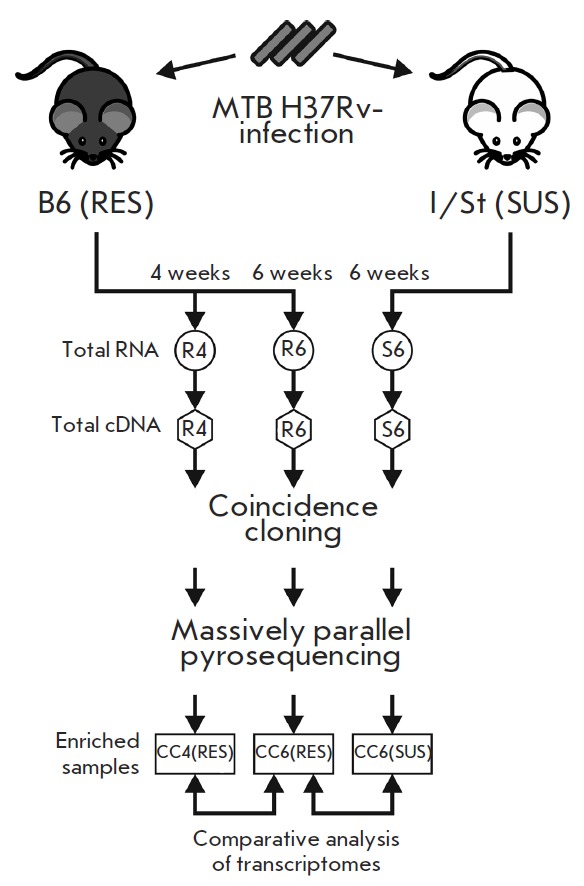
Scheme of transcriptome comparison. RES – genetically resistant mice, SUS – genetically susceptible mice, CC – library enriched in bacterial cDNA

**Noncoding RNAs**

The recent studies employing the methods of massively parallel sequencing have demonstrated that the bacterial transcriptome is much more complex than it was thought to be. The noncoding RN As have been shown to be an important part of the transcriptome and to regulate various cell functions (replication, energy metabolism, and regulation of the expression of virulence factors in a number of pathogenic bacteria) [11].

We have searched for transcripts from the loci lying in the intergenic regions, since such localization attests to the fact that these transcripts have the potential to belong to the class of noncoding RN As. Based on data on the structural organization of the genome, we have identified 3,069 intergenic regions. The analysis of pyrosequencing data showed that the transcripts of 164 (5.3%), 221 (7.2%), and 356 (12.3%) intergenic regions were presented in the CC 4(RE S), CC 6(SUS), and CC 6(RE S) samples, respectively. . It should be mentioned that transcripts from 27 (0.9%) of the intergenic loci were present in each of the three samples, while transcripts from 2,490 (81.1%) loci have not been observed.

The experimental data on the expression of intergenic regions were compared with the available literature and database information on the localization of small RN A genes.

A significant expression of sequences from a number of intergenic regions (in particular, from IGR3987 and IGR0629 in sample CC 6(SUS) and from IGR1186 in sample CC 6(RE S)) was detected. The experimental results confirmed the presence of expression from the intergenic regions IGR3987, IGR0629, and IG1136, which correlates with the data obtained by Arnvig *et al*. [12] (according to the denotations used in [12], the intergenic regions IGR2975, IGR0479, and IGR0858, respectively). We believe that the detected expression of intergenic sequences can attest to the potential localization of small RN A genes in these loci. With allowance for their differential expression, it could be an indication of the compensatory response of a pathogen to external factors.

**Genes whose expression increases with infection progression**

We compared the transcriptomes of *M. tuberculosis* during the infection progression in the genetically resistant to tuberculosis mouse strain (СС6(RE S) and CC 4(RE S)) and at a single time point in the genetically different mouse strains (СС6(RE S) and CC 6(SUS)). The comparison was aimed at searching for genes whose expression increases with infection progression (i.e., in B6 mice on week 6 after the infection) as compared to the other conditions. The comparison of СС6(RE S) and CC 4(RE S) revealed 226 genes whose expression increased with infection progression in the tissues of B6 mice. The comparison of СС6(RE S) and CC 6(SUS) revealed 253 genes with increased expression in the CC 6(RE S) sample.

The comparison of CC 6(RE S) and CC 4(RE S) revealed only 17 genes whose expression in CC 6(RE S) was higher than that in CC 4(RE S), while the expression of 44 genes in CC 6(RE S) was higher as compared to CC 6(SUS). These results presumably demonstrate that the first comparison characterizes the dynamics of the changes in pathogen gene expression with time within a single micro-environment. In the latter case, the differences between two different micro-environments are revealed, which affects a larger number of genes whose expression increases in CC 6(RE S).

The genes whose expression in the CC 6(RE S) sample is increased only as compared to CC 4(RE S) mostly fall into the following categories: cell wall and cell processes, intermediary metabolism and respiration, and lipid metabolism. The protein products of 12 out of 17 genes were previously detected in the fraction containing the cellular membrane and/or cell wall, where they predominantly play roles in transport and protection. For instance, the *embA *gene encodes indolylacetylinositol arabinosyltransferase EmbA, which participates in arabinan synthesis. Mutations in this gene cause resistance to ethambutol. The *Rv3273 *gene encodes carbonate dehydratase, which is involved in sulfate transport (TubercuList). An analysis using the KEGG Pathways (http://www.genome.jp/kegg/pathway.html) and TBCYC (http://tbcyc.tbdb.org/) databases has revealed no metabolic pathways that would be activated at the later stages of infection progression. This fact can either result from random fluctuations in pathogen gene expression, its response to random changes in the properties of the micro-environment, or it can be an indication of small, but significant changes in the functional activity of *M. tuberculosis *at different time points.

The comparison of the CC 6(RE S) and CC 6(SUS) samples has revealed that CC 6(RE S) contains a larger number of genes whose expression is higher in this sample as compared to that in CC 6(SUS). The increased energy metabolism manifested itself as increased expression of the genes encoding three NADH-dehydrogenase subunits (*nuoH, nuoI, nuoL*); a higher activity of the tricarboxylic acid cycle (*acn*); and as increased expression of the *Rv1916 *gene. *Rv1916 *is the second component of the *aceA *(*icl2*) gene, which is divided in the genome of *M. tuberculosis *H37Rv into two individually expressed modules, *Rv1915 *and *Rv1916 *(*aceAa *and* aceAb*). The other important differences include the increased expression of the genes whose products are responsible for the metabolism and catabolism of lipids and amino acids (*lipV, lipF, Rv2531c*), as well as the enzymes that participate in DNA repair (*recO, recB*). This is a rather predictable pattern, since the microenvironment of the resistant host is a hostile habitat, which explains the demand for higher activity from the repair systems. The increased expression of lipolytic enzymes (*lipF, lipV, plcA*), enzymes of the tricarboxylic acid cycle, and *aceAb *may attest to the fact that a greater amount of lipids is used as a source of energy and carbon.

We focused on searching for *M. tuberculosis *genes whose increased expression does not depend on the genetic features of the host organism. These genes form a certain basic set, whose genes are responsible for the universal compensatory response of the pathogen to adverse environmental conditions. These genes are referred to as commonly upregulated genes (CU G): a total of 209 genes whose expression was increased in both comparisons (*Table 2*). According to the results of transposon mutagenesis, 44 genes of *M. tuberculosis* H37Rv are essential [13]. Rv3569c, Rv3537, and Rv3563 were earlier shown to be essential for survival of *M. tuberculosis *in mouse macrophages (TubercuList, http://tuberculist.epfl.ch).

**Table 2:** GUC gene family

Gene	Functional category (according to TubercuList)
Rv0028, Rv0074, Rv0269c, Rv0274, Rv0281, Rv0421c, Rv0428c, Rv0433, Rv0448c, Rv0455c, Rv0492A, Rv0525, Rv0597c, Rv0695, Rv1179c, Rv1186c, Rv1203c, Rv1232c, Rv1419, Rv1428c, Rv1828, Rv1835c, Rv1868, Rv1998c, yfiH, Rv2974c, Rv3030, Rv3031, Rv3205c, Rv3272, Rv3519, Rv3627c, Rv3651, Rv3662c, Rv3703c, Rv3753c, Rv0026, Rv0061, Rv0140, Rv0141c, Rv0145, Rv0332, Rv0712, Rv0785, Rv0998, Rv1514c, Rv1515c, wbbL2, Rv1760, Rv2077A, Rv2135c, Rv2466c, Rv2699c, Rv2751, Rv2823c, Rv3067, Rv3090, Rv3094c, Rv3510c	CH – conserved hypotheticals
Rv0051, Rv0309, lprL, Rv0621, Rv0876c, lytB2, irtA, Rv1687c, secA2, Rv2209, Rv2265, mmpL7, Rv3194c, Rv3658c, embC, espE, ponA1, Rv0072, narK3, iniA, cpsY, lpqR, pstS1, Rv0996, kdpC, Rv1097c, sugB, Rv1431, Rv1667c, Rv2136c, Rv2203, efpA, rip, Rv2963, lpqF	CWaCP – cell wall and cell processes
Rv0161, ndhA, Rv0526, menH, Rv0805, lipU, glyA1, dapE, atpF, atpH, Rv1432, frdB, cmk, plcD, lipJ, cobK, cobS, cysK1, cysE, gdh, gabT, miaA, ilvC, guaB2, cyp142, hsaD, Rv0089, Rv0331, aspC, hemA, Rv0567, atsA, gltA2, Rv0943c, Rv1096, Rv1106c, narH, thrB, hisB, ilvG, rocD1, plcB, phoH1, ggtB, lepA, Rv2499c, dapF, purU, kstD, folP1	IMaR – intermediary metabolism and respiration
end, fusA1, polA, lysX, helZ, spoU, ppiB, thrS, Rv3201c	IP – information pathway
Rv0095c, Rv0920c, Rv2791c	ISaP – insertion sequences and phages
fadD10, nrp, fadD7, fadE4, fadD2, fadD12, pks17, pks12, mbtF, mbtE, mbtC, TB7.3, accA3, fadE27, fadD17, accD4, mmaA3, mmaA1, fadE19, Rv2613c, fadD26, ppsC, ppsD, fadD19, fadE31, fadE32, pks13	LM – lipid metabolism
PPE8, PE_PGRS19, PE16, PPE34, PPE50, PE2, PPE64	PE/PPE – PE/PPE protein families
pknA, senX3, trcR, Rv1359, fhaA, Rv0465c, Rv3066, Rv3736	RP – regulatory proteins
Rv2645, Rv2818c	U – unknown
treS, mce2C, Rv1026, ephB, vapB16, Rv2581c, vapC3, cinA, virS	VDA – virulence, detoxification, adaptation

We have grouped the GUG genes into functional categories (TubercuList) and compared their distribution to that of all the *M. tuberculosis *genes. These distributions show a general similarity, with the exception of the genes that belong to the lipid metabolism category, which can further demonstrate their importance for the processes of bacterial adaptation.

Two categories (conserved hypotheticals (59 genes) and unknown (2 genes) contained slightly less than a third of all the genes. Despite the absence of any known functions, the genes in this category are potential therapeutic targets, since their low degree of homology with the genes of other microorganisms means that they are typical of mycobacteria (in particular, of* M. tuberculosis*); thus, they presumably determine their virulence properties.

The high expression level of the genes of various nutrient uptake and accumulation systems (e.g., phosphate (*pstS1*) and iron (*irtA, mbtC, mbtE, mbtF*)) attests to the fact that mycobacteria exist under conditions of insufficient nutrient supply. Phosphorus deficiency is also indicated by the increased expression of the *senX3* gene, the sensor component of the two-component regulatory system *senX3*/*regX3*, which regulates the so-called stringent response under conditions of phosphorus deficiency. The transition to the use of lipids as the main source of energy and carbon is indicated by the expression of lipid metabolism genes (*fadD, fadE, lipU, lipJ*). Another feature of the genes that belong to the CU G set consists in increased expression of genes whose products are somehow associated with amino acid metabolism (*aspC, hisB, thrB, thrS*). The reason behind this phenomenon remains unclear, since stimulation of enzyme expression can be caused by both the absence of the required amino acids (and, hence, the demand for their synthesis) and by their presence (and the possibility to use them by bacteria).

The transition to anaerobic nitrate respiration, which is typical of latent infection, is attested by the increased expression of the *narH *and *narK3 *genes [14]. We have also classified the *atpF *and *atpH *genes as CU G genes, although decreased expression of these genes during infection progression is reported, since the energy demand of a pathogen decreases as it acquires the state of latent infection [15, 16].

The function of PE/PPE proteins is not clear. They are considered to be required to ensure antigenic variability in mycobacteria [17]. Nevertheless, the expression levels of the *Rv0152c *and *Rv0355c *genes in the CC 6(RE S) sample are high, and their expression has also been detected in CC 4(RE S) and CC 6(SUS). The* Rv3135 *gene belongs to the group of essential genes in* M. tuberculosis *H37Rv. All these observations suggest that PE/PPE genes have some other functions in addition to ensuring antigenic variability.

Finally, the *secA2*gene is worth mentioning. This gene encodes translocase SecA2, the component of the* M. tuberculosis *Sec transport system that enables the secretion of superoxide dismutase SodA and catalase KatG. A live vaccine based on the *ΔsecA2 *mutant of* M. tuberculosis *has demonstrated high efficiency and safety in tests on animals [18].

A microarray-based comparative analysis of the expression profiles of 17 members of the *M. tuberculosis* complex in activated and nonactivated murine macrophages has recently been conducted[19]. As a result, 280 genes (168 with universally increased and 112 genes with universally decreased expression) were identified whose changes in expression were independent of the strain and the activation status of a macrophage. We compared the CU G genes with 168 genes from [19] characterized as having universally increased expression and selected eight genes *(Rv0140, Rv0145, atsA, Rv2466c, fadD26, ilvC, Rv3067, *and* kstD*) that were featured in both lists. Such an insignificant coincidence can be attributed to the fact that a) the infection of cultured macrophages is a relatively simplified model as compared to the complex relationships between the host cells and the pathogen during the infectious process in the host organism; b) the data obtained by a microarray analysis of expression can significantly differ from those obtained by massively parallel sequencing. In particular, Ward *et al*. [20] reported a divergence of the results obtained using these two methods.

Nevertheless, the results obtained by us and Homolka* et al. *[19] are not widely divergent. A functional analysis of the genes with increased expression has demonstrated that they are associated with intracellular stress factors, such as hypoxia and reactive oxygen and nitrogen species, cell wall remodeling, and fatty acid metabolism. The genes associated with iron deficiency (genes in the *mtbA-F *cluster) and those involved in the biosynthesis of valine and isoleucine (*ilvB–ilvN– ilvC*) and phthiocerol dimycocerosates (PDIM) of the cell wall (*ppsA-D*) can be mentioned as an example.

**Products of the CUG family genes as potential therapeutic targets**

Six genes which could potentially be used as therapeutic targets (or have already been proposed as such) were selected after a review of the available literature and databases. The products of these genes (*hisB, aspC, PPE50, Rv1026, ilvC*, and *Rv1186c*) have been mentioned as attractive therapeutic targets, since the disturbances in their functional activity has the maximum destabilizing effect on the metabolism of *M. tuberculosis*. Thus, for example, aspartate aminotransferase AspC encoded by the *aspC (Rv0337c) *gene was identified in the metabolic network of a mycobacterial cell as an enzyme whose inactivation affects a large number of other *M. tuberculosis *proteins, thus efficiently disintegrating a large number of biochemical cycles [21]. The protein products of two other genes, *Rv1186c *and PPE50 (*Rv3135)*, have been included in the list of the most promising therapeutic targets, which was compiled based on data on the expression, participation in various metabolic pathways, and structural homology with other bacterial and human proteins [22]. The* Rv1026, hisB (Rv1601)*, and *Rv3001c *genes are believed [23] to encode products suitable for designing specific inhibitors. The protein encoded by the *Rv1601 *(*hisB*) gene is independently considered to be a promising therapeutic target [24]. It should be mentioned that the expression level of the *Rv1026 *gene encoding pyrophosphatase is increased in the macrophages and lungs of infected mice [25, 26], as well as under conditions of inhibited translation in mycobacteria [27]. It has recently been demonstrated that polyphosphate deficiency due to the hydrolytic activity of Rv1026 can change the fatty acid content in the cell wall of *M. smegmatis*, thus affecting sliding motility and biofilm formation [28]. Hence, it can be expected that the products of the remaining CU G genes could be used to design therapeuticals or for diagnosing tuberculosis.

## CONCLUSIONS

Infectious diseases caused by intracellular pathogenic bacteria pose a serious medical problem. The progression of the infection depends not only on host defense mechanisms, but also on the specific expression of bacterial genes. Changes in the gene expression in response to various reactions of the host immune system are necessary for the survival and reproduction of pathogenic bacteria. The investigation of the changes in the transcription profile of *M. tuberculosis *in response to various stimuli and external factors allows one to describe the adaptative mechanisms required for an efficient infection of a host organism by a bacterium.

The investigation of the transcription profiles of *M. tuberculosis *under various conditions made it possible to reveal the gene set (CU G) whose expression increases with infection progression and is independent of the genetic features of the host organism. The expression of genes from this core set can be regarded as the universal response of mycobacteria to various environmental stress factors. Further accumulation and analysis of* M. tuberculosis *gene expression data will considerably simplify the development of efficient approaches to the diagnosis and treatment of tuberculosis.

## Abbreviations

PDIM: phthiocerol dimycocerosate
CC: coincidence cloning
CUG: commonly upregulated genes
